# Deletions in the pyruvate pathway of *Salmonella* Typhimurium alter SPI1-mediated gene expression and infectivity

**DOI:** 10.1186/2049-1891-4-5

**Published:** 2013-02-25

**Authors:** Jason Abernathy, Carolina Corkill, Carolee Hinojosa, Xianyao Li, Huaijun Zhou

**Affiliations:** 1 Department of Animal Science, University of California, 95616, Davis, CA, USA; 2 Department of Poultry Science, Texas A&M University, 77845, College Station, TX, USA; 3 College of Animal Science and Technology, Shandong Agricultural University, 271018, Taian, Shandong, China

**Keywords:** *adhE*, Metabolism, *pflB*, Pyruvate, *Salmonella* pathogenicity island, *Salmonella* Typhimurium, Virulence

## Abstract

**Background:**

*Salmonella enterica* serovar Typhimurium is a major foodborne pathogen worldwide. *S.* Typhimurium encodes type III secretion systems via *Salmonella* pathogenicity islands (SPI), producing the major effector proteins of virulence. Previously, we identified two genes of *Salmonella* pyruvate metabolism that were up-regulated during chicken cell infection: pyruvate formate lyase I (*pflB*) and bifunctional acetaldehyde-CoA/alcohol dehydrogenase (*adhE*). We were therefore interested in examining the role these genes may play in the transmission of *Salmonella* to humans.

**Methods:**

Mutant strains of *Salmonella* with single gene deletions for *pflB* and *adhE* were created. Invasion and growth in human HCT-8 intestinal epithelial cells and THP-1 macrophages was examined. Quantitative PCR was performed on 19 SPI-1 genes.

**Results:**

In HCT-8 cells, both mutant strains had significantly higher intracellular counts than the wild-type from 4 to 48 h post-infection. Various SPI-1 genes in the mutants were up-regulated over the wild-type as early as 1 h and lasting until 24 h post-infection. In THP-1 cells, no significant difference in internal *Salmonella* counts was observed; however, SPI-1 genes were largely down-regulated in the mutants during the time-course of infection. We also found five SPI-1 genes - *hilA*, *hilC hilD*, *sicP* and *rtsA* - which were up-regulated in at least one of the mutant strains in log-phase broth cultures alone. We have therefore identified a set of SPI-1 virulence genes whose regulation is effected by the central metabolism of *Salmonella*.

## Background

*Salmonella* serovars cause an array of diseases, ranging from gastroenteritis to systemic infections [1,2]. While there are thousands of characterized serovars, few have been demonstrated as virulent to humans [3,4]. *Salmonella enterica* serovar Typhimurium is one such strain, with a broad spectrum of hosts including pig, chicken and humans [1,5,6]. *S.* Typhimurium can be ubiquitous in the environment and within certain animal reservoirs; it can act as a commensal, produce an asymptomatic infection, and/or trigger disease in various hosts [7]. Serovar Typhimurium thus remains one of the top infectious food-borne pathogens worldwide.

The most common route of entry for *S.* Typhimurium is by oral transmission. Once contact is made with a human host through this route, the bacterium initiates infection by invading intestinal epithelial cells using a type III secretion system (T3SS) [8-10]. This system is encoded by a 40 kilobase region of the genome called *Salmonella* pathogenicity island (SPI) 1 [11-18]. The T3SS of *S.* Typhimurium consists of structural components (i.e. needle complex) that make up the apparatus used to inject effector proteins into the cytoplasm of host cells [12,19-21]. The effector proteins then promote infection by altering the physiology of the host cell. Specifically, *Salmonella* can seize the host cell machinery and induce rearrangements in the actin cytoskeleton and cause alterations in signal transduction that helps facilitate further systemic infection [22-24]. In mammals, the intracellular phase of infection proceeds in a coordinated effort with an additional type III secretion system encoded by SPI-2 [25-27]. Overall, the T3SS-1 encoded by SPI-1 promotes invasion of host intestinal epithelial cells [28,29], initiation of inflammation [27,30,31], and intracellular survival and persistence [32-34], while TTSS-2 encoded by SPI-2 is needed to promote intracellular growth [35-39].

While the T3SS and its genetic regulators are a central component in the ability of *Salmonella* to establish a systemic infection, the capability of *Salmonella* to invade and persist in a host has been partly linked to the central metabolism of the bacterium (reviewed in [40]) and environmental cues [41-44]. Alterations in *Salmonella* metabolic pathways, such as glycolysis and the tricarboxylic acid (TCA) cycle, using both *in vitro* and *in vivo* models have demonstrated the importance of factors outside of the T3SS in its pathogenicity [40]. For instance, deletions in the pyruvate dehydrogenase subunit E1 (*aceE*) gene of *S.* Enteritidis has been shown to attenuate the strain in a chicken model [45]. In murine models, the loss of functional phosphofructokinase (Pfk) activity prevents growth of *S*. Typhimurium [46], while deletions in other TCA cycle genes hyper-activate the mutants in macrophages though interestingly attenuates the response in BALB/c mice [47]. As the SPI genes and the T3SS have been extensively studied as key pathogen modulators in *Salmonella*, determining the ability of additional metabolic genes to affect pathogenicity remains to be explored.

**Figure 1:**
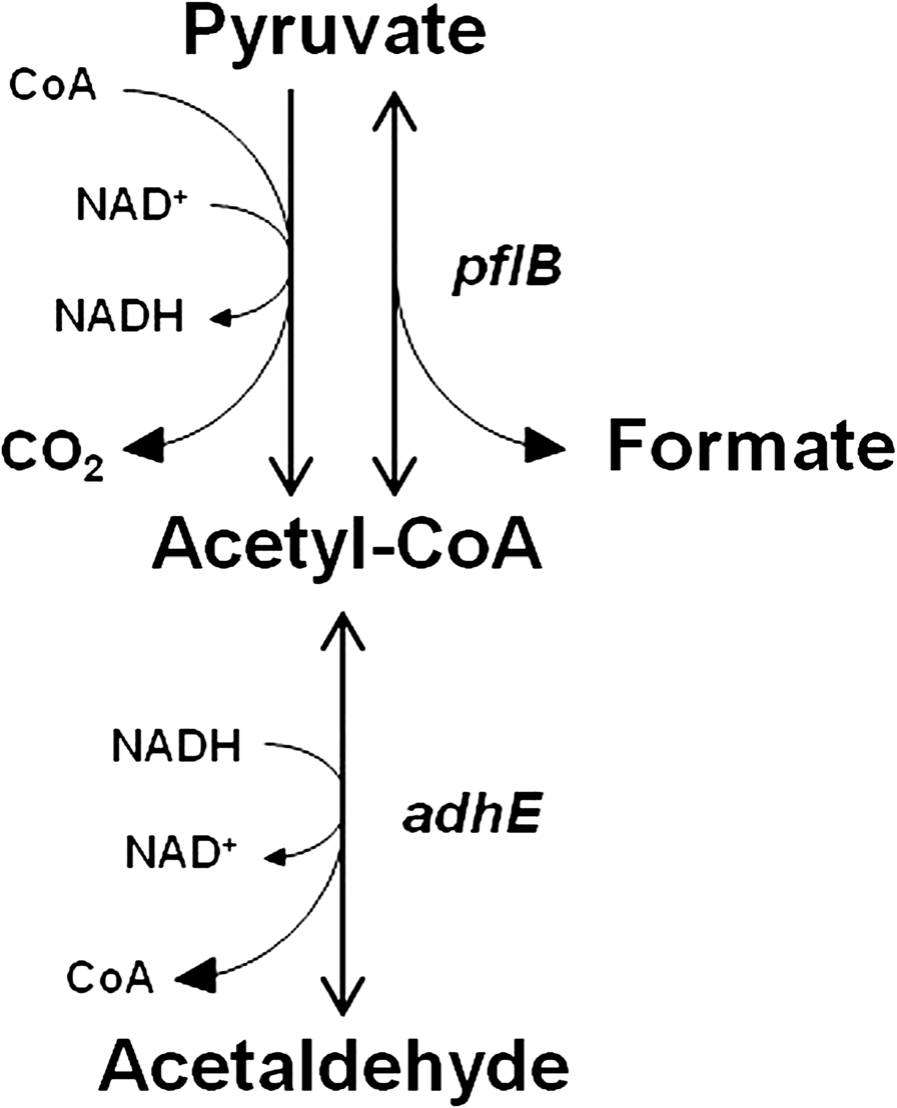
**A simplified model of the pyruvate metabolism pathway in *Salmonella *Typhimurium.** Genes producing metabolic enzymes (in italics) were deleted in the creation of mutant strains, including pyruvate formate lyase I (*pflB*) and bifunctional acetaldehyde-CoA/alcohol dehydrogenase (*adhE*).

In our previous study, we measured global gene expression of the *Salmonella enterica* serotype Typhimurium genome in chicken heterophils (data not shown). Two *Salmonella* genes, pyruvate formate lyase I (*pflB*) and bifunctional acetaldehyde-CoA/alcohol dehydrogenase (*adhE*), were significantly up-regulated after infection. Since these genes are not typical virulence genes but play a role in cellular metabolism (Figure 1), we sought to understand their role during infection. Therefore, the overall aim of this study is to further characterize the importance these *Salmonella* genes play in its pathogenesis. Deletion mutants of these two genes were created, and gentamicin protection assays and quantitative PCR of SPI-1 genes were performed on human epithelial and macrophage cell lines. The resulting experiments show that these two genes of the pyruvate metabolic pathway affect both infectivity of epithelial cells and SPI-1 gene expression.

## Methods

### Creation of *Salmonella* mutants

The *Salmonella enterica* serovar Typhimurium ATCC 14028 strain was used as the wild-type in this study. This strain was also used in the construction of mutant gene knock-out isolates. Two genes, pyruvate formate lyase I (*pflB*) and bifunctional acetaldehyde-CoA/alcohol dehydrogenase (*adhE*), produce enzymes of the pyruvate metabolic pathway as illustrated (Figure 1). To assess the effects of these genes during invasion, two mutant strains, Δ*pflB::kan* (*S.* Typhimurium gene number STM0973) and Δ*adhE::kan* (*S.* Typhimurium gene number STM1749), were created by the lambda red recombination method with adaptations as described by Santiviago et.al. [48]. Gene-specific primers used for the creation of each isolate are listed in Table 1. The recombination event was confirmed by polymerase chain reaction (PCR) using primers to the flanking genomic regions.

**Table 1:** **A list of the *Salmonella *primers used in this study**

**Gene**	**Forward sequence (5**^**′**^**-3**^**′**^**)**	**Reverse sequence (5**^**′**^**-3**^**′**^**)**	**Locus or reference**	**Technique**
*pflB*	GAAGGTAGGTGTTACATGTCCGAGCTT AATGAAAAGTTAGCCACAGTGCAGGCT GGAGCTGCTTC	TTTCAGTCAAACCCATTACAT GGTCTGCGTGAAGGTACGAGT AATCATATGAATATCCTCCTTAG	STM0973	Lambda red
*adhE*	TATCAGGAGAGCATTATGGCTGTTACT AATGTCGCTGAACTTAACGTGCAGGCT GGAGCTGCTTC	AAAACAGATAACGAATTAAGC GGATTTTTTCGCTTTTTTCTC TGCCATATGAATATCCTCCTTAG	STM1749	
16S rRNA	AGGCCTTCGGGTTGTAAAGT	GTTAGCCGGTGCTTCTTCTG	[49]	qPCR
*hilA*	ATAGCAAACTCCCGACGATG	ATTAAGGCGACAGAGCTGG	[50]	
*hilC*	CTCACCTCTTCAGCGGCCAGT	CACCCGCAAATGGTCACAGGCT	STM2867	
*hilD*	GGTAGTTAACGTGACGCTTG	GATCTTCTGCGCTTTCTCTG	[50]	
*invC*	TCGGTCGATCGCTGCAA	CCCTGGTCGCGAAAATATTC	STM2894	
*invE*	CGAATGACGCCAGCTGTTC	TGCGTCAGGCGTCGTAAA	STM2897	
*invF*	TGAAAGCCGACACAATGAAAAT	GCCTGCTCGCAAAAAAGC	STM2899	
*invH*	GGTGCCCCTCCCTTCCT	TGCGTTGGCCAGTTGCT	STM2900	
*invJ*	CGCCGCCGGTTAATTG	GCTTCCGCTGCAACCAA	STM2892	
*orgA*	AGGCAGGGAGCCTTGCTT	CCCTGATGCATTGCCAAAA	STM2870	
*orgB*	ACCATCCCGAAACGCTTTTA	TTGCCCCTCAGGCTTATCG	STM2869	
*prgH*	TGAACGGCTGTGAGTTTCCA	GCGCATCACTCTGACCTACCA	STM2874	
*prgJ*	GGTCTATGGAAACGGACATTGTC	CGCCGAACCAGAAAAAGC	STM2872	
*prgK*	GGGTGGAAATAGCGCAGATG	TCAGCTCGCGGAGACGATA	STM2871	
*rtsA*	ACCCGTGGTGAGCTTGATGAGT	CCTGTCCAGGTGGGGAGCAT	SEE_03946	
*sicA*	GATGAGTCTCTGCGGGCAAA	GCTCTGTCTCCGCCGTTTT	STM2886	
*sicP*	AGATGATATCTGGTTATTGAACGGTATG	CTGCCGCCAGATAGAATCG	STM2879	
*sipA*	CGTCTTCGCCTCAGGAGAAT	TGCCGGGCTCTTTCGTT	STM2882	
*sipB*	GGCGCGCTGCTAACCAT	TCGCCCCACCGGTAAAA	STM2885	
*sipC*	ATCAGGCTGGTCGATTTACG	GTACGCCGCTACTCAGGAAC	[50]	

### Cell culture

Human cell lines HCT-8 and THP-1 were used in this study. HCT-8 is an ileocecal colorectal adenocarcinoma epithelia cell line, while THP-1 is a monocytic leukemia cell line that can be stimulated to differentiate into macrophages. HCT-8 was grown using RPMI-1640 with 2.05 mmol/L L-Glutamine media (HyClone, Thermo Scientific, Rockford, IL) supplemented with 10% fetal bovine serum (Atlanta Biologicals, Lawrenceville, GA), 1% of 100 mmol/L sodium pyruvate (Thermo Scientific), and 1% of 10 000 U penicillin/10 mg streptomycin per mL (Thermo Scientific). THP-1 was grown in suspension using RPMI-1640 with 2.05 mmol/L L-Glutamine media (HyClone) supplemented with 10% fetal bovine serum (Atlanta Biologicals), 1% of 10,000 U penicillin/10 mg streptomycin per mL (Thermo Scientific), and 1.75 μL of 14.3 mM β-Mercaptoethanol (Sigma-Aldrich, St. Louis, MO). Both cell lines were cultured at 37°C in an air-jacketed incubator (NuAire, Plymouth, MN) with 5% CO_2_ and constant humidity.

### Invasion assays

The infectivity of *Salmonella* on both HCT-8 and THP-1 was defined as the number of internal *Salmonella* cells per viable human cell at each unit of time. Internal *Salmonella* counts on both cell lines were determined using a gentamicin protection assay (GPA). Viable human cells were assayed by trypan blue exclusion. Invasion assays on each cell line were performed in at least three independent experiments, using triplicate wells at each time-point. Probabilities for statistical significance were calculated using Student’s *t*-test.

For HCT-8 cells, 2 × 10^5^ cells were seeded in 12-well plates with 1 mL complete media (without antibiotics) and grown until confluent. Overnight ATCC 14028, Δ*pflB::kan*, and Δ*adhE::kan* strains of *Salmonella* grown at 37°C in Luria-Bertani (LB) broth were sub-cultured for 3 h in pre-warmed (37°C) LB broth before challenge to ensure log-phase cultures. HCT-8 cells were challenged with each *Salmonella* strain at a multiplicity of infection (MOI) of 100 by replacing media with *Salmonella* infectious media. Immediately upon challenge, plates were centrifuged at 500 × g for 5 min and placed in a CO_2_ incubator and allowed to incubate for 30 min. After this time, cells were washed with phosphate buffered saline (PBS, pH 7.4) and infectious media was replaced with 1 mL complete media supplemented with 100 μg/mL gentamicin sulfate (Mediatech, Manassas, VA). After 30 min, media was replaced with 2 mL complete media supplemented with 10 μg/mL gentamicin sulfate (Mediatech) and kept through the duration of the study. The first time-point of 1 h post-infection was collected by washing the infected HCT-8 cells with PBS and lysing with 1 mL PBS + 1% Triton X-100 (Sigma-Aldrich). Internalized *Salmonella* were then counted by serial dilutions on pre-warmed LB agar in triplicate. At the same time, infected HCT-8 cells were counted on a TC10 automated cell counter (Bio-Rad, Hercules, CA) using a 1:1 ratio of cells to 0.4% trypan blue dye (Bio-Rad). Additional time-points of 4, 18, 24, and 48 h post-infection were also collected.

For THP-1 cells, 8 × 10^5^ THP-1 cells were seeded in 12-well plates with 1 mL complete media (without β-Mercaptoethanol or antibiotics) and 200 nmol/L phorbol 12-myristate 13-acetate (PMA; Millipore, Billerica, MA) for 24 h to allow differentiation of suspension monocytes to adherent macrophages. After 24 h, media was exchanged with pre-warmed media (without β-Mercaptoethanol or antibiotics) omitting PMA and cells were allowed to develop for an additional 24 h. Overnight cultures from ATCC 14028, Δ*pflB::kan*, and Δ*adhE::kan* strains of *Salmonella* were sub-cultured for 2.5 h in pre-warmed LB broth and then incubated with 10% human AB serum (Mediatech) for 30 min prior to challenge. THP-1 cells were challenged 48 h after seeding at a MOI10 from each *Salmonella* strain, and invasion was assessed of similarly as with HCT-8 challenge. To assess the role that opsonization may play on infectivity using these *Salmonella* strains, separate GPA experiments were also performed where strains were not incubated with human AB serum prior to challenge. Time-points of 1, 2.5, 4, and 6 h post-infection were collected from non-opsonized challenge experiments. Lower time-points and MOI were selected for non-opsonized challenge as the THP-1 cells were largely damaged and apopotic at MOI 100 and with longer incubations (data not shown), and has previously been observed [51].

### Quantitative PCR

Real-time reverse-transcription quantitative PCR (RT-qPCR) was performed to assess mRNA expression levels of 19 selected SPI-1 genes. The primers used for RT-qPCR are listed in Table 1. SPI-1 transcripts were measured from both *Salmonella* grown in cultures and after invasion of host cells.

To generate complementary DNA (cDNA) for RT-qPCR from *Salmonella* cultures, 5 mL of ATCC14028, Δ*pflB::kan*, and Δ*adhE::kan* strains were grown overnight at 37°C with agitation, then sub-cultured 1:25 in pre-warmed LB broth for 4 h. The three strains were equalized by OD_600_ spectrophotometry and RNA was isolated by the RNeasy Protect Bacteria Kit (Qiagen, Valencia, CA) according to the manufacturer’s protocol. Potential contaminating genomic DNA was removed by the TURBO DNA-free™ kit (Ambion, Carlsbad, CA), as directed by the manufacturer. Reverse transcription reactions were then performed by the ThermoScript™ RT-PCR System for First-Strand cDNA Synthesis (Invitrogen, Carlsbad, CA) from 4 μg total RNA from each sample, using the supplied random hexamers as RT-primers, in 20 μL total volume reactions. Reactions for each sample were diluted 1:10 with sterile nanopure water and used as templates for RT-qPCR.

To generate cDNA for RT-qPCR from *Salmonella* after invasion of host cells, HCT-8 and THP-1 cells were again infected with ATCC 14028, Δ*pflB::kan*, or Δ*adhE::kan* strains by the GPA in additional independent experiments, with the following modifications. At each time-point post infection, cells were washed with PBS and lysed directly by the addition of 400 μL TRI-reagent (Ambion) to each well and gentle pipetting. Total RNA was then extracted using the RiboPure™-Bacteria Kit (Ambion) according to the manufacturer’s protocol. The TURBO DNA-free™ kit (Ambion) was used to remove potential genomic DNA contamination. Total RNA from each sample (representing one well of a 12-well plate) was quantified using a spectrophotometer (NanoDrop 1000, Wilmington, DE) and equimolar amounts from each of three replicate wells were pooled. RT-reactions were performed using the ThermoScript™ RT-PCR System for First-Strand cDNA Synthesis (Invitrogen) from 800 ng total RNA from each sample, using the supplied random hexamers as RT-primers, in 20 μL total volume reactions. Reactions for each sample were diluted 1:5 with sterile nanopure water and used as templates for RT-qPCR.

All RT-qPCR reactions were performed using the *Power* SYBR Green PCR Master Mix (Applied Biosystems, Foster City, CA), with modifications. A total reaction volume of 10 μL was used, to include: 5 μL of 2X SYBR Green, 2 μL of [1 μmol/L] forward primer, 2 μL of [1 μM] reverse primer, and 1 μL template cDNA. Quantitative PCR was performed on an ABI 7900HT system (Applied Biosystems), with cycling and dissociation curve parameters used according to the recommendations from the manufacturer. Each RT-qPCR reaction was performed using at least three technical replicates for each sample. After real-time PCR cycling, each product was assessed for quality and specificity by the corresponding dissociation curve. Raw data was edited using the SDS v2.4 Software (Applied Biosystems), and cycle threshold (Ct) values were recorded. Ct values were uploaded into the Relative Expression Software Tool (REST2009; Qiagen) [52] for comparisons. Data was considered significant at *p*-value < 0.05 after permutation testing using randomizations. The standard error of the mean was estimated from confidence intervals.

## Results

### *Salmonella* invasion of HCT-8 epithelial cells

**Figure 2:**
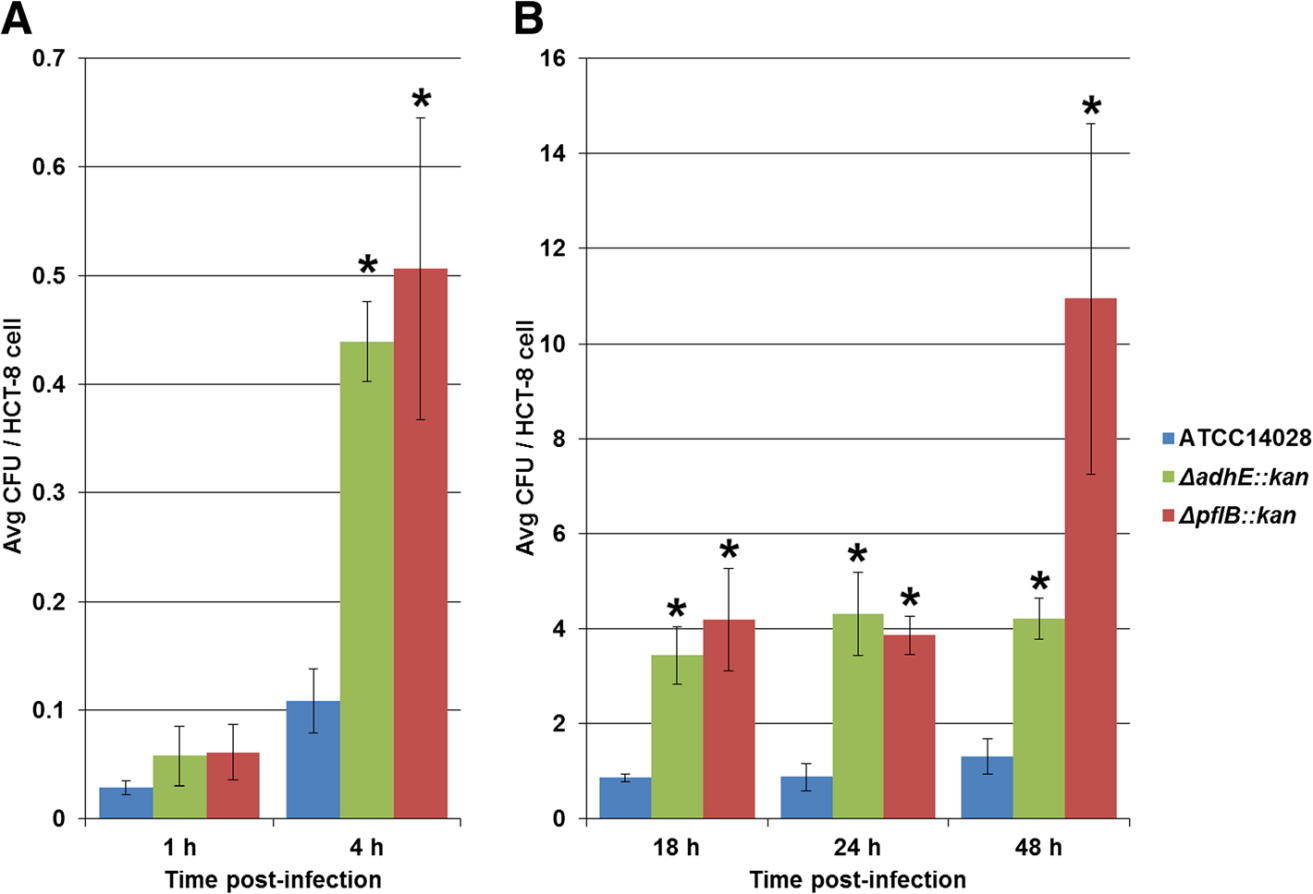
**Invasion of HCT-8 cells by *Salmonella *Typhimurium strains. (A)** Time-points of 1 h and 4 h post-infection; **(B)** Time-points of 18 h, 24 h, and 48 h post-infection. The X-axis represents time (h) while the Y-axis represents average colony forming units (CFU) of bacteria per viable HCT-8 cell. The different *Salmonella* strains are color-coded according to the legend. Asterisk indicates significance of the mutant strain from the wild-type at *p* < 0.01 assessed at each time.

After HCT-8 cells were challenged with either *Salmonella* wild-type or mutant strains, the number of intracellular bacteria as assessed by counting colony forming units (CFUs) on LB agar were averaged from repeated independent GPA experiments. At the earliest time post-infection of 1 h, there was no significant difference in average CFUs between the wild-type and mutant strains (Figure 2A). However, by 4 h, there was a significant increase in average CFUs in the mutant strains, Δ*pflB::kan* and Δ*adhE::kan*, compared to the wild-type strain, ATCC 14028 (Figure 2A). At 4 h, there were approximately 4–5 CFU of either mutant per 10 HCT-8 cells, while ATCC 14028 remained near 1 CFU/10 HCT-8 cells, thus representing an approximate 4–5 fold increase in *Salmonella* mutants over the wild-type. Increased numbers of mutant strains over the wild-type observed at 4 h post-infection also lasted through the duration of the study at 48 h post-infection, measured at increments of 18, 24, and 48 h (Figure 2B). At 18 h post-infection, there were 4.2 and 3.9 CFU per HCT-8 cell from Δ*pflB::kan* and Δ*adhE::kan* strains, respectively, while the wild-type ATCC 14028 strain remained at near 1 CFU/cell, thus representing an approximate 4-fold significant difference. Similarly at 24 h post-infection, there were 3.9 and 4.3 CFU per viable epithelial cell from Δ*pflB::kan* and Δ*adhE::kan* strains, respectively, while the wild-type again remained around 1 CFU/HCT-8 cell at this time-point. The wild-type strain ATCC 14028 was mostly consistent in the level of infectivity and growth measured by GPA with an average of 6.4 CFU per 10 HCT-8 cells over the 48 h course of study, ranging from 3 CFU/100 HCT-8 cells at 1 h to 1.3 CFU/HCT-8 cell at 48 h post-infection. However, in the mutant strains there was an approximate 8-fold significant increase in the number of CFUs between the 1 h and 4 h time-points and, likewise, an approximate 8-fold increase between the 4 h and 18 h time-points. Average CFU/HCT-8 cell was also increased in the mutants, with an average of 3.9 and 2.5 CFU/HCT-8 cell in Δ*pflB::kan* and Δ*adhE::kan*, respectively. CFUs of both mutant strains remained consistent between 18 h and 24 h post-infection; however, at 48 h post-infection a spike in Δ*pflB::kan* was observed (Figure 2B). At 4 h through 48 h post-infection, internal growth of the mutants was found to be highly significant (*p*-value < 0.01). Overall there was either a steady or an upward trend in the numbers of internalized *Salmonella* through the 48 h course of infection in epithelial cells with the magnitude of replication being significantly greater by the mutant strains beginning a 4 h post-infection (Figure 2).

### *Salmonella* invasion of THP-1 macrophage cells

**Figure 3:**
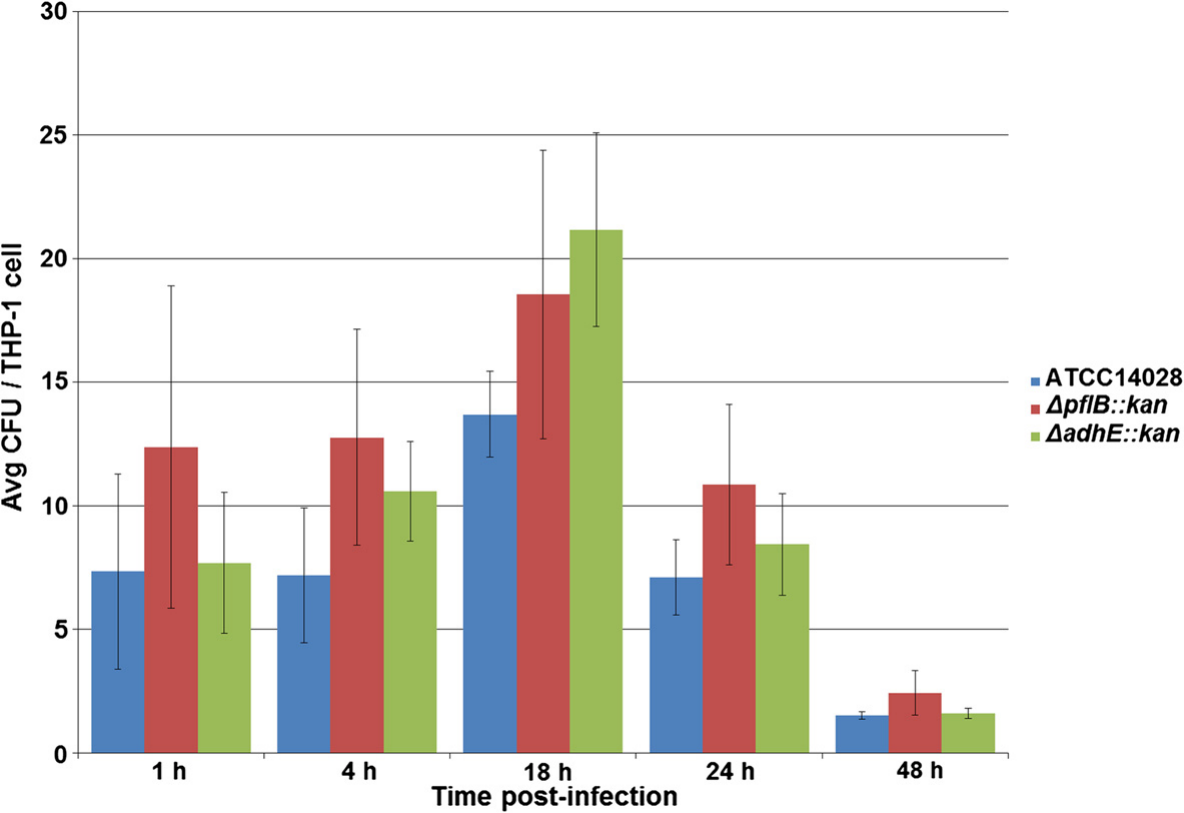
**Invasion of THP-1 cells by opsonized *Salmonella *Typhimurium strains.** Time-points of 1, 4, 18, 24, and 48 h post-infection are shown. The X-axis represents time (h) while the Y-axis represents average colony forming units (CFU) of bacteria per viable THP-1 cell. The different *Salmonella* strains are color-coded according to the legend.

For challenge of THP-1 cells with *Salmonella* strains opsonized by treatment with human AB serum, time increments were chosen the same as with HCT-8 challenge. Again, average numbers of internalized *Salmonella* strains were assessed by counting CFUs. The numbers of internalized *Salmonella* ranged from approximately 1.5 to 21 CFU per viable THP-1 cell (Figure 3). ATCC 14028 wild-type strain had an average of 7.4 CFU/THP-1 cell, Δ*pflB::kan* strain had an average of 11.4 CFU/THP-1 cell, and Δ*adhE::kan* strain had an average of 9.9 CFU/THP-1 cell over the course of the 48 h study. There was a general trend that the mutant strains, in particular Δ*pflB::kan*, had higher CFUs per differentiated THP-1 cell than ATCC 14028. However, no significant difference in average CFU counts was observed between either of the mutant or wild-type strains after repeated GPA experiments. This was the case at all of the five time-points tested, from 1 h to 48 h post-infection (Figure 3). Overall, there was a bell-curved trend in the numbers of internalized *Salmonella* throughout this study, where bacteria peaked at 18 h post-infection, and then began to decline as the viable THP-1 cells declined (Figure 3).

**Figure 4:**
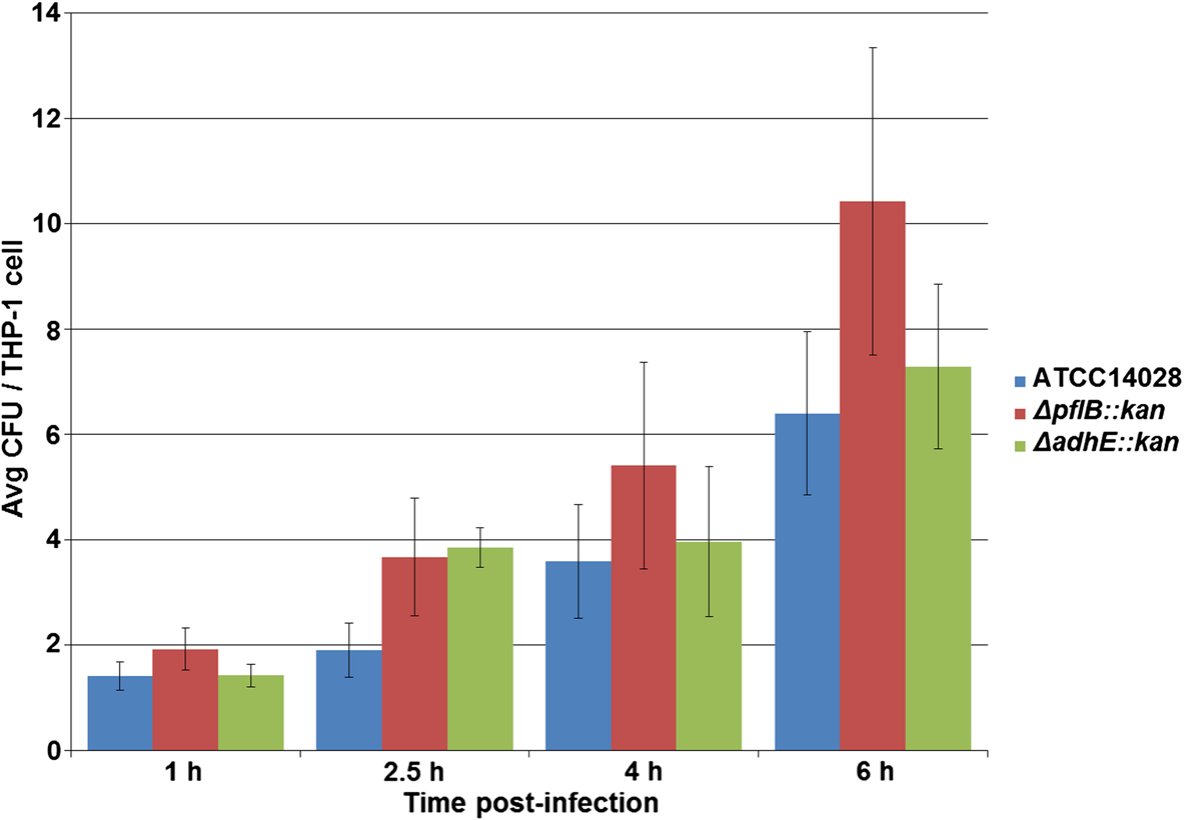
**Invasion of THP-1 cells by non-opsonized *Salmonella *Typhimurium strains.** Time-points of 1, 2.5, 4, and 6 h post-infection are shown. The X-axis represents time (h) while the Y-axis represents average colony forming units (CFU) of bacteria per viable THP-1 cell. The different *Salmonella* strains are color-coded according to the legend.

For challenge of THP-1 cells with non-opsonized *Salmonella* strains, time-points of 1, 2.5, 4, and 6 h post-infection were selected to assess infectivity. The numbers of internalized *Salmonella* cells ranged from approximately 1.4 to 10.4 CFU per viable THP-1 cell (Figure 4). ATCC 14028 wild-type strain had an average of 3.3 CFU/THP-1 cell, Δ*pflB::kan* strain had an average of 5.4 CFU/THP-1 cell, and Δ*adhE::kan* strain had an average of 4.1 CFU/THP-1 cell over the course of the 6 h study. There was a general trend that the mutant strains, in particular Δ*pflB::kan*, had higher CFUs per differentiated THP-1 cell than ATCC 14028. Again however, no significant difference in average CFU counts was observed between either of the mutant or wild-type strains after repeated GPA experiments. This was the observation at any of the four time-points tested, from 1 h to 6 h post-infection (Figure 4). Overall, there was an upward trend in the numbers of internalized *Salmonella* throughout this study as viable THP-1 cells had not yet become noticeably apoptotic (Figure 3).

After the THP-1 GPA trials, a direct comparison was made between 1 h and 4 h post-infection to assess the role opsonization may play between the *Salmonella* parent and mutant strains. For the opsonized challenge, ATCC 14028 had an average internalization of 7.3 and 7.2 CFU/THP-1 cell at 1 h and 4 h, respectively. Δ*pflB::kan* had an average internalization of 12.4 and 12.8 CFU/THP-1 cell at 1 h and 4 h, respectively. Δ*adhE::kan* had an average internalization of 7.7 and 10.6 CFU/THP-1 cell at 1 h and 4 h, respectively (Figure 3). For the non-opsonized challenge, ATCC 14028 had an average internalization of 1.4 and 3.6 CFU/THP-1 cell at 1 h and 4 h, respectively. Δ*pflB::kan* had an average internalization of 1.9 and 5.4 CFU/THP-1 cell at 1 h and 4 h, respectively. Δ*adhE::kan* had an average internalization of 1.4 and 4.0 CFU/THP-1 cell at 1 h and 4 h, respectively (Figure 4). Thus, larger average CFU counts were observed by challenge of THP-1 cells with opsonized bacteria.

### SPI-1 gene expression in culture

**Figure 5:**
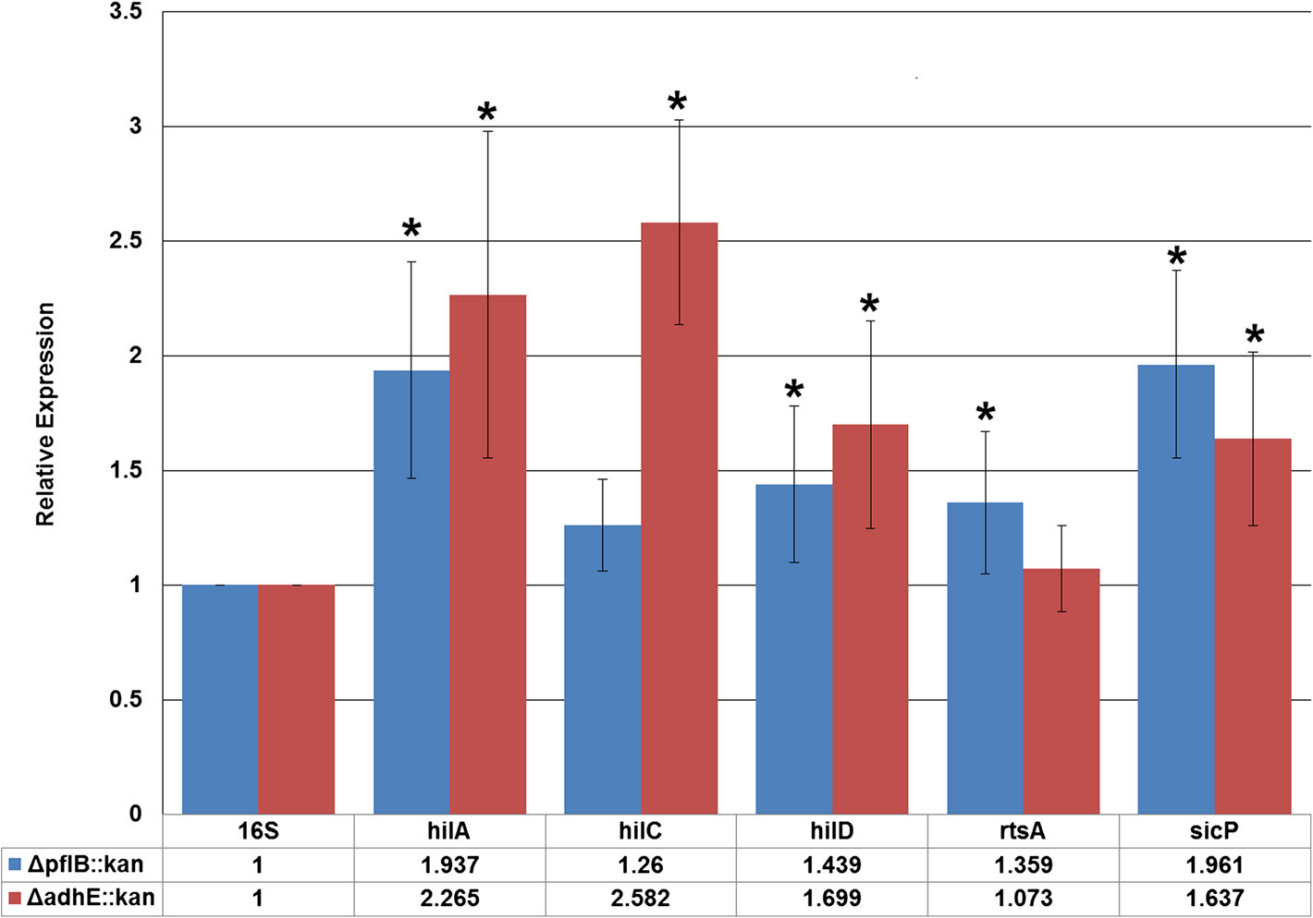
**Quantitative RT-PCR of *Salmonella *Typhimurium strains from cultures.** Gene expression of select SPI-1 invasion genes measured by RT-qPCR from *Salmonella* mutants Δ*pflB::kan* and Δ*adhE::kan* and comparing expression to the wild-type strain. The 16S rRNA gene was used for normalization. Genes are listed along the X-axis while relative expression level is listed along the Y-axis. Asterisk indicates significance of the mutant strain from the wild-type at *p* < 0.05 assessed for each gene.

To determine if the significant increase in numbers of *Salmonella* mutants to HCT-8 cells was potentially due to the type III secretion system, gene expression was measured in 19 SPI-1 genes (Table 1) by qPCR and compared to the expression in the wild-type strain. Differential regulation was measured from SPI-1 transcripts in log-phase cultures. A total of five genes were found significantly up-regulated in at least one of the mutant strains (Figure 5). In the Δ*pflB::kan* strain, *hilA*, *hilD*, *rtsA*, and *sicP* genes were up-regulated compared to ATCC 14028. Relative expression values ranged from 1.36 to 1.96 over the wild-type in genes found significant by qPCR (*p* < 0.05). In the Δ*adhE::kan* strain, *hilA*, *hilC*, *hilD*, and *sicP* genes were up-regulated compared to ATCC 14028. Relative expression values ranged from 1.64 to 2.58 over the wild-type in genes found significant by qPCR (Figure 5).

### SPI-1 gene expression after invasion of HCT-8 epithelial cell line

Since a set of SPI-1 genes were up-regulated in cultures alone, we were interested to determine if a larger response involving a greater number of genes or a greater magnitude of the fold-change was observed through interaction with epithelial cells. The response of SPI-1 after infection with HCT-8 cells was determined by using qPCR on the same panel of 19 genes. SPI-1 genes were found to be up-regulated over the wild-type as early as 1 h post-infection (Table 2).

**Table 2:** **Quantitative RT-PCR of *Salmonella *during invasion of HCT-8 cells**

**Time post-infection**	**Strain**	***Salmonella *gene**	Expression*	Regulation**
1 h	Δ*pflB::kan*	*hilC*	1.28	UP
		*hilD*	1.60	
		*prgJ*	1.48	
		*prgK*	1.92	
		*sicA*	1.31	
	Δ*adhE::kan*	*hilC*	1.26	
		*invE*	3.16	
		*invH*	1.56	
		*orgB*	1.25	
		*prgJ*	1.60	
		*prgK*	1.96	
		*sicA*	1.15	
4 h	Δ*pflB::kan*	*rtsA*	2.52	UP
		*sicA*	1.64	
		*sipA*	1.88	
	Δ*adhE::kan*	*hilA*	3.12	
		*orgA*	3.70	
		*orgB*	1.66	
		*rtsA*	2.47	
		*sicA*	1.67	
		*sicP*	1.56	
		*sipA*	1.62	
18 h	Δ*pflB::kan*	*hilD*	2.15	UP
		*prgH*	1.85	
		*prgJ*	3.08	
		*sicA*	2.78	
		*sipA*	1.88	
		*sipC*	3.21	
	Δ*adhE::kan*	*invC*	1.61	
24 h	Δ*pflB::kan*	*prgH*	−1.92	DOWN
		*prgJ*	−2.43	
		*prgK*	−1.75	
		*sicP*	−3.41	
		*sipA*	−3.36	
		*sipB*	−3.39	
	Δ*adhE::kan*	*invC*	−2.66	
		*sipA*	−2.07	

*Values listed are relative fold-change values as compared to the wild-type strain ATCC 14028; **All genes listed are significant at *p*-value < 0.05.

In Δ*pflB::kan*, five SPI-1 genes were up-regulated at 1 h, ranging from 1.28 to 1.92 fold change over the wild-type. At 4 h, three SPI-1 genes were up-regulated with relative expression values ranging from 1.64 to 2.52 fold over the wild-type. At 18 h post-infection, six SPI-1 genes were found to be up-regulated, with fold changes of 1.85 to 3.21 over the wild-type. In Δ*adhE::kan*, seven SPI-1 genes were up-regulated at 1 h post-infection, with relative expression values ranging from 1.15 to 3.16 fold over the wild-type. At 4 h, seven SPI-1 genes were up-regulated with relative expression values ranging from 1.62 to 3.70 fold over the wild-type. At 18 h post-infection, *invC* was up-regulated 1.61 fold in Δ*adhE::kan* over ATCC 14028. By 24 h post-infection, SPI-1 genes were either down-regulated in both mutant strains, or not significantly different from the wild-type strain.

### SPI-1 gene expression after invasion of THP-1 macrophage cell line

*Salmonella* invasion gene expression from bacteria invaded into differentiated macrophages, THP-1 cell line, was also examined for non-opsonized wild-type and mutant strains. Similarly to non-opsonized *Salmonella* GPA, time-points of 1, 2.5, 4, and 6 h post-infection were chosen for testing of SPI-1 gene expression by qPCR.

In strain Δ*pflB::kan*, several SPI-1 genes were differentially expressed between the mutant and wild-type (Table 3). At 1 h, there was no significant differential gene expression. By 2.5 h, 6 SPI-1 genes were down-regulated with expression values ranging from −1.34 to −1.70, while *invE* was up-regulated 1.7-fold over the wild-type at the same time. At 4 h, *hilA*, *hilD*, *invF*, and *prgJ* were slightly up-regulated, with expression values of 1.18 to 1.72 fold over the wild-type. The largest degree of differential expression was observed at 6 h post-infection. A total of 17 out of the 19 SPI-1 genes tested (89%) were down-regulated, ranging from −1.3 to −2 fold change over the wild-type.

**Table 3:** **Quantitative RT-PCR of non-opsonized *Salmonella *strain Δ*pflB::kan *during invasion of THP-1 cells**

**Time post-infection**	***Salmonella *gene**	Expression*	Regulation**
1 h	No change		
2.5 h	*invE*	1.71	UP
	*invC*	−1.46	DOWN
	*invH*	−1.41	
	*rtsA*	−1.70	
	*sicA*	−1.52	
	*sicP*	−1.34	
	*sipC*	−1.36	
4 h	*hilA*	1.72	UP
	*hilD*	1.36	
	*invF*	1.19	
	*prgJ*	1.18	
6 h	*hilA*	−1.61	DOWN
	*hilC*	−1.97	
	*hilD*	−1.55	
	*invC*	−1.54	
	*invE*	−1.51	
	*invF*	−1.52	
	*invH*	−1.61	
	*orgA*	−1.66	
	*orgB*	−1.30	
	*prgH*	−1.43	
	*prgJ*	−1.46	
	*rtsA*	−1.83	
	*sicA*	−1.89	
	*sicP*	−1.63	
	*sipA*	−1.58	
	*sipB*	−2.00	
	*sipC*	−1.70	

*Values listed are relative fold-change values as compared to the wild-type strain ATCC 14028; **All genes listed are significant at *p*-value < 0.05.

In strain Δ*adhE::kan*, SPI-1 gene expression was down-regulated at every time-point examined (Table 4). At 1 and 2.5 h post-infection, all genes tested were significantly down-regulated as compared to ATCC 14028. At 4 h post-infection, 14 of the 19 SPI-1 genes tested (74%) were down-regulated, with *prgJ* having the lowest magnitude of the fold-change at −1.67 and *sipB* having the highest magnitude of the fold-change at −4.44 under the wild-type. At 6 h post-infection, 16 of the 19 SPI-1 genes tested (84%) were down-regulated, with *orgB* having the lowest magnitude of the fold-change at −1.31 and *sipB* having the highest magnitude of the fold-change at −3.58 under the wild-type.

**Table 4:** **Quantitative RT-PCR of non-opsonized *Salmonella *strain Δ*adhE:kan *during invasion of THP-1 cells**

**Time post-infection**	***Salmonella *gene**	Expression*	Regulation**
1 h	All genes tested were significantly down-regulated		
2.5 h	All genes tested were significantly down-regulated		
4 h	*hilC*	−2.53	DOWN
	*invC*	−2.63	
	*invE*	−2.46	
	*invF*	−2.14	
	*invH*	−1.98	
	*orgB*	−1.71	
	*prgH*	−1.79	
	*prgJ*	−1.67	
	*prgK*	−1.91	
	*rtsA*	−2.58	
	*sicA*	−3.91	
	*sicP*	−1.73	
	*sipB*	−4.44	
	*sipC*	−2.91	
6 h	*hilA*	−2.08	DOWN
	*hilC*	−2.76	
	*hilD*	−1.56	
	*invC*	−2.76	
	*invE*	−2.07	
	*invF*	−2.44	
	*invH*	−2.30	
	*orgA*	−2.36	
	*orgB*	−1.31	
	*prgH*	−1.71	
	*rtsA*	−2.33	
	*sicA*	−3.31	
	*sicP*	−1.72	
	*sipA*	−2.22	
	*sipB*	−3.58	
	*sipC*	−2.28	

*Values listed are relative fold-change values as compared to the wild-type strain ATCC 14028; **All genes listed are significant at *p*-value < 0.05.

## Discussion

SPI-1 of *Salmonella enterica* serovars is an important virulence factor that has been well-studied in several animal models and in disease outbreaks [53,54]. SPI-1-mediated infectivity of *Salmonella* Typhimurium was examined by creation of null mutations in genes that were up-regulated in our previous host-pathogen microarray study (data not shown). Those *Salmonella* genes, pyruvate formate lyase I (*pflB*) and bifunctional acetaldehyde-CoA/alcohol dehydrogenase (*adhE*), play a major role in the pyruvate metabolism of the organism (Figure 1). Here we show that these two genes can also affect virulence and SPI-1 gene expression.

For the HCT-8 intestinal epithelial cell line, internalized mutant *Salmonella* CFUs were significantly increased over the wild-type at 4 h post-infection and lasted through 48 h post-infection at which time the experiment was terminated (Figure 2). Since the T3SS encoded by SPI-1 is canonically involved in early contact with non-phagocytic cells such as fibroblasts and intestinal epithelia [27,30,31], a selected group of SPI-1 genes (Table 1) in mutant *Salmonella* were measured by qPCR and compared to the wild-type expression. Several SPI-1 genes were up-regulated after HCT-8 cell challenge, from 1 h post-infection lasting to 24 h post-infection when SPI-1 expression in the mutants decreased compared to the wild-type (Table 2). These up-regulated genes included *hilC*, *hilD*, *rtsA*, *sipA*, - all major genes involved in the early response to infection [55]. Some SPI-1 genes were up-regulated at multiple times post-infection and in both mutants, such as *sicA*, a gene encoding a complex chaperone protein required for *Salmonella* entry into host cells [56,57]. Overall, the results indicated that increased internalized growth of strains of *S.* Typhimurium deficient for transcripts of the pyruvate pathway in HCT-8 cells may in part be due to a differential regulation of genes in the T3SS.

Additional evidence in supporting this notion included qPCR results on SPI-1 genes in log-phase cultures alone. We found that the major regulators of SPI-1 were up-regulated when compared to the wild-type. These included *hilA* and *hilD* in both of the mutant strains, and *hilC* and *rtsA* in at least one of the mutant strains (Figure 5). HilA is the major regulator of SPI-1 invasion [41]. The *hilA* gene encodes an OmpR/ToxR family transcriptional regulator that activates the expression of invasion genes in response to both environmental and genetic factors [41]. Further, the proteins encoded by *rtsA*, *hilC* and *hilD* bind to a DNA region upstream of *hilA* and induce *hilA* expression [58-60]. Therefore, these genes could be expected to be induced at an early stage, as they are key modulators of invasive *Salmonella* infection and *hilD* appears to bridge communication between SPI-1 and SPI-2 regulation (reviewed in [55]). These results further indicate the increased virulence of the mutant strains to HCT-8 is occurring via a differential regulation of genes involved in the T3SS. Interestingly, the *sicP* gene was also up-regulated in both mutant strains measured from cell culture alone. SicP serves as a chaperone for the SPI-1 effector *Salmonella* protein tyrosine phosphatase (SptP). SptP has been shown to have several functions in the establishment of *Salmonella* infection, in both epithelial cells and macrophages [61,62]. While the gene expression of SptP was not examined, the up-regulation of the chaperone *sicP* transcripts could indicate early formation of SicP-SptP complexes even before contact with a host, as these two genes appear to be transcriptionally coupled [63]. The full significance of the gene expression response of *sicP* to alterations in the pyruvate metabolism pathway remains to be further explored.

We further examined the effects of the mutant strains on the infectivity of THP-1 macrophage cell line. We observed no significant difference in the internalization of *Salmonella* mutants over the wild-type strain, whether the invasion assays were using opsonized or non-opsonized bacteria (Figures 2 &3). Since we were interested in addressing the effects of our mutations on SPI-1-dependent virulence, we chose to examine SPI-1 gene expression using invasion assays with non-opsonized bacteria. Interestingly, while no significant internalization between strains was observed, SPI-1 gene expression was mostly down-regulated in the mutants after a challenge of THP-1 cells (Tables 3 &4). During the systemic phase of macrophage infection by *Salmonella*, SPI-1 expression is typically repressed to allow internal replication of the bacterium and suppress excess apoptosis [64]. Therefore, as differentiated THP-1 are phagocytic-type cells and infection is largely established via SPI2-encoded T3SS, low SPI-1 expression of invaded macrophages could therefore be expected based on previous efforts [65-70].

The specific *Salmonella* knockout genes and the role they may play in its pathogenesis were further examined. Our data supports the overall results of Huang et al. [50] that a *pflB* mutation in *Salmonella* can effect SPI-1 gene expression as measured from cultures alone. In addition to the previous work [50], we identified multiple SPI-1 genes that are differentially expressed both in cultures and at several time-points post-infection of an *in vitro* challenge using human HCT-8 cells. Knockout of the *pflB* gene likely produces an imbalance of metabolites in which the presence mimics the conditions in the host gastrointestinal tract [50]. We further show that another gene of the *Salmonella* pyruvate pathway (*adhE*) can have a similar effect on intestinal epithelia, suggesting that gut metabolites in addition to formate and/or other feedback mechanisms due to environmental cues may be at work. As AdhE is a multifunctional enzyme, another possibility for increased invasion of a Δ*adhE* mutant in addition to an SPI1-mediated virulence mechanism could be formulated. A lack of *AdhE* appears to stimulate type I fimbrial adhesion [71,72], important adhesions on the surface of *Salmonella* that may mediate attachment and internalization of bacteria [73]. Further experimentation would be warranted to determine the exact mechanism and co-factors involved with the pyruvate pathway involvement in *Salmonella* virulence.

For the generation of mutant strains, each recombination event was confirmed by PCR using primers to the flanking genomic regions. These confirmations, in addition to the evidence provided in this study, give confidence in the specificity of our deletions. By using lambda-red for site-specific mutagenesis, evidence suggests any potential downstream effects are reduced or eliminated with this system over that of random mutagenesis/transposons, including issues of polarity (e.g. [48]). We do acknowledge however that complementation assays to restore the phenotype could provide additional evidence, but these were not performed here.

We also acknowledge the role the host microbiome plays in *Salmonella* pathogenicity [74], particularly since microbiota may contribute to pyruvate pathway metabolites utilized in the establishment of infection [50]. The mammalian gastrointestinal tract contains high levels of short chain fatty acids as the result of the breakdown of foodstuffs by the digestive processes and the action of resident bacteria [75]. We therefore would be highly interested in future *in vivo* studies to help determine the mechanisms involved in the interplay between the host genetics, the gut environment, and the microbiome on *Salmonella* pathogenesis when dealing with mutant strains produced through metabolic pathway mutations.

## Conclusions

Deletions of two genes, *pflB* and *adhE*, involved in the pyruvate metabolism pathway of *Salmonella* Typhimurium were shown to alter SPI-1 gene expression. In bacterial cell culture alone, five SPI-1 genes from the two mutant strains were up-regulated from the wild-type. In a challenge with human HCT-8 and THP-1 cell lines, SPI-1 gene expression was also significantly altered from the wild-type at various times post-infection. We therefore have identified a set of SPI-1 genes that crosstalk between *Salmonella* pyruvate metabolism and virulence. This work provides a further framework for examining the role that central metabolism plays in the infection of *Salmonella* Typhimurium.

## Competing interests

The authors declare that they have no competing interests.

## Authors’ contributions

JA and XL designed assays and performed the necessary experiments to generate *Salmonella* mutants. JA, CC and CH performed cell culture and gene expression experiments. HZ conceived, planned and guided this study. JA and HZ drafted the manuscript. All authors read and approved the final manuscript.
